# Guidewire entrapment in the Chiari network during the insertion of a hemodialysis catheter: a case report

**DOI:** 10.1186/s12882-024-03618-7

**Published:** 2024-07-29

**Authors:** Ming Ye, Dongying Xiang, Liang Li, Yinggang Qin, Yibing Zhu, Long He, Chuan Guo

**Affiliations:** 1 Department of Nephrology, Guang’anmen Hospital, China Academy of Chinese Medical Sciences, Beijing, China; 2 Department of Ultrasound, Guang’anmen Hospital, China Academy of Chinese Medical Sciences, Beijing, China; 3 Department of Oncology, Guang’anmen Hospital, China Academy of Chinese Medical Sciences, Beijing, China; 4 Department of Emergency, Guang’anmen Hospital, China Academy of Chinese Medical Sciences, Beijing, China

**Keywords:** Chiari network, Hemodialysis catheter, Catheterization, Right heart system

## Abstract

**Background:**

The Chiari network, a remnant of fetal anatomy, consists of a mesh-like structure within the right atrium. With advancements in cardiac interventions, complications associated with the Chiari network have increasingly been reported. However, there are few reports about guidewire or catheter entrapment in the Chiari network during the insertion of a dialysis catheter.

**Case presentation:**

A 46-year-old male with end-stage renal disease was hospitalized and underwent a digital subtraction angiography-assisted catheterization of the right internal jugular vein tunnel-cuffed dialysis catheter. When the guide wire entered a depth of about 20 cm, it was difficult to advance, manifested as resistance when twisting the guide wire and inability to enter the inferior vena cava. After the peelable sheath was inserted, it was difficult to pull out the guide wire. After repeated attempts to rotate the guide wire, the guide wire was finally pulled out. A fibrous tissue was wrapped around the tip of the guide wire. Its length was 6 cm, with a smooth surface and tough texture. We considered that the tissue we pulled out was most likely a part of a Chiari network.

**Conclusions:**

This case highlights the potential for the Chiari network to complicate surgical procedures, including difficulty with guidewire and catheter manipulation. Attention should be paid to Chiari networks. Echocardiography can be used to identify the Chiari network. During the surgery, forcefully pulling out a stuck guidewire is not suggested, to avoid the risk of tearing the atrial wall and causing pericardial tamponade. An urgent consultation with ultrasound doctors and cardiac surgeons might be helpful in such cases.

## Background

**Fig. 1:**
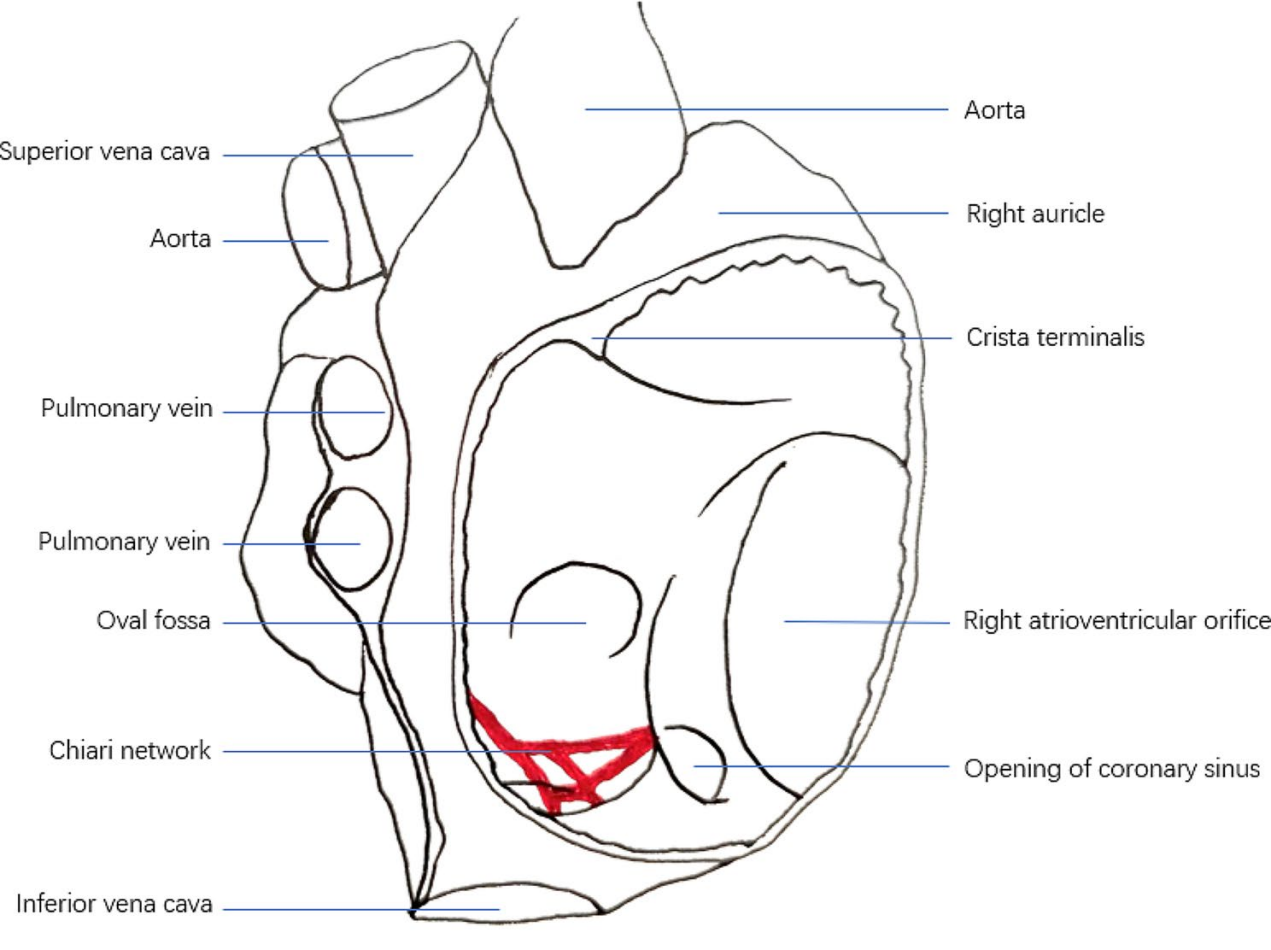
An anatomical diagram of the Chiari network

The Chiari network is an anatomic variant formed by the residual strands of the right valve of the sinus venosus, often presenting as a fenestrated membrane or a reticular network within the valve of the inferior vena cava and coronary sinus (Fig. 1) [1, 2]. The Chiari network is associated with a variety of clinical conditions including thromboembolism, infective endocarditis, cardiac arrhythmias, patent foramen ovale, flow obstruction, and atrial septum defects [2]. The incidence of the Chiari network is about 2%, and usually, there are no clinical symptoms or signs [3]. However, during the examination or treatment of the right heart system, such as the implantation of a right heart catheter or pacemaker, the Chiari network may cause significant operational difficulties in inserting or exiting the guide wire or catheter [4–12]. In severe cases, cardiac surgery may be necessary to remove the catheter or guidewire [11]. With the development of cardiac interventions, complications associated with the Chiari network have increasingly been reported [2]. However, there are few case reports about guidewire or catheter entrapment in the Chiari network during the insertion of a dialysis catheter, which may be related to the limited understanding of the Chiari network by nephrologists [13]. In this study, we report a case of guidewire entrapment in the Chiari network during the hemodialysis catheter insertion. An informed consent statement was obtained for this study.

## Case presentation

A 46-year-old male with a decade-long history of proteinuria and diagnosed with end-stage renal disease was admitted for hemodialysis catheter placement. His serum creatinine was 1303µmol/L on the day before admission and 1382µmol/L on the first day of admission. Previously, his last test for serum creatinine was four years ago, and the result was 88µmol/L. The ultrasound suggested diffuse changes in both kidneys. On the day of hospitalization, the patient underwent a digital subtraction angiography (DSA) assisted catheterization of the right internal jugular vein tunnel-cuffed dialysis catheter.

**Fig. 2:**
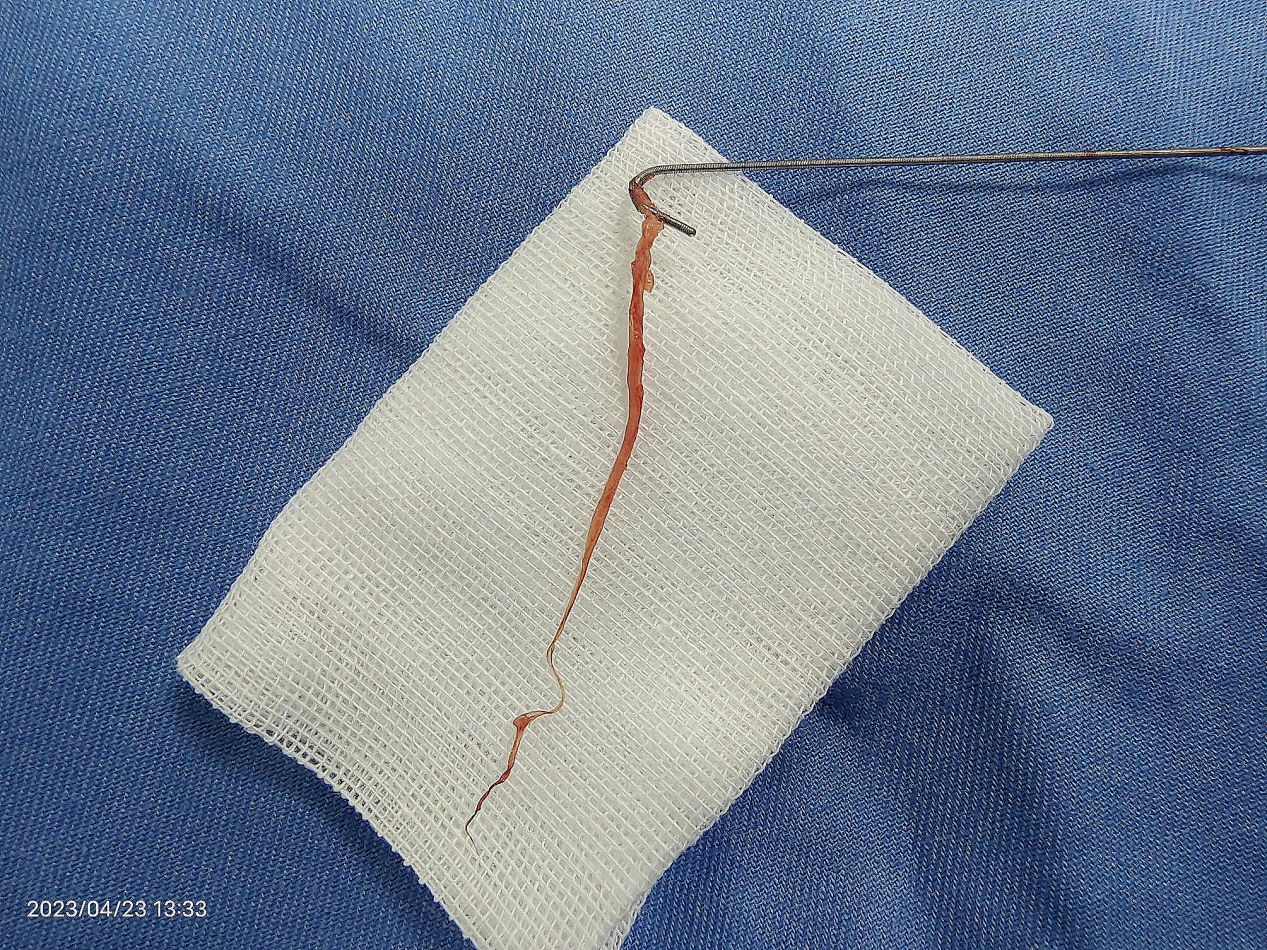
The figure showed the fibrous tissue wrapped around the tip of the guidewire

The following was the situation during the operation. In the beginning, a J-shaped spring coil guidewire was inserted under ultrasound guidance through the right internal jugular vein. When the guide wire entered a depth of about 20 cm, it was difficult to advance, manifested as resistance when twisting the guide wire and inability to enter the inferior vena cava. At that time, the DSA image showed that the tip of the guide wire was in the right atrium, and there was no arrhythmia observed in electrocardiogram monitoring, indicating that the tip of the guide wire did not touch the atrial wall. Although the guide wire failed to enter the inferior vena cava, considering that the tip of the guide wire was in the right atrium, which met the conditions for catheter insertion, the surgery continued. Then we inserted the peel-able sheath along the guide wire. Afterward, it was difficult to pull out the guide wire, so we rotated the guide wire and attempted to pull it out. At this point, the DSA image showed that the guide wire was stuck at the level of the right atrium. After repeated attempts to rotate the guide wire, the guide wire was finally pulled out. A fibrous tissue was wrapped around the tip of the guide wire. Its length was 6 cm, with a smooth surface and tough texture (Fig. 2). After the operation, we performed an echocardiography to detect remnants of the Chiari network. No remnant was found.

## Discussion and conclusions

Central venous dialysis catheters are one of the common hemodialysis pathways for end-stage renal disease patients. Mechanical complications may occur during the insertion, including guidewire entrapment [14, 15]. This may cause vascular or organ damage, guidewire breakage, and distal embolism. Therefore, a comprehensive understanding of the complications of catheterization is necessary for surgeons. The incidence of the Chiari network is about 2%, usually found by postmortems and echocardiography examinations [3]. There have been many reported cases of entrapment of the catheters or leads by the Chiari networks [4–12]. However, it is not widely known to nephrologists, as far as we know [16]. Therefore, we assume that some Chiari networks causing difficulties in guidewire placement have not been identified and reported during the operations of hemodialysis catheterization.

We experienced a case of a guidewire entangled by fibrous tissue. Although a Chiari network was not detected through the transthoracic echocardiography after the operation, we considered that the guidewire was entrapped with a Chiari network for the following reasons. First, we pulled out a Chiari network-like tissue wrapped with the guidewire. The tissue was almost the same as the images in other case reports. Second, we consulted with the cardiovascular surgeons, they considered it was most likely a Chiari network. Since there was no arrhythmia observed when the guidewire failed to enter the inferior vena cava, we considered that the tip of the guidewire did not touch the atrial wall. And the fibrous tissue we pulled out was previously located in the right atrium. Third, we thoroughly reviewed the literature and reports on difficulties and complications during the operations of the right heart system. Based on our maximum knowledge and comprehensive judgment, we believed that the tissue we pulled out was most likely part of a Chiari network.

Our case suggests the importance of recognizing the Chiari network as a potential risk factor for difficulties and complications during the insertion of the right heart system, including hemodialysis catheters, pacemakers, and other types of central venous catheters. There are limitations to our work. First, the fibrous tissue specimen was not sent for pathology. A microscopic examination of the obtained tissue might help conclude about the origin of the tissue. Based on the literature review, in right heart system intervention surgery, the fibrous membrane-like substance wrapped around the front end of the guidewire was commonly found to be proliferative fibrous tissue in pathological examination and considered a Chiari network. In our case, the tissue we pulled out was identified as fibrous tissue based on its morphology and texture, but no pathological examination provided further evidence. Second, the patient did not undergo an echocardiography examination before surgery. Postoperative echocardiography did not show any residual Chiari network, which may be related to the residual Chiari network adhering to the wall and unclear ultrasound display after spatial structural damage. Third, the pulled-out fibrous tissue was only a part of the Chiari network and did not display a typical network structure. However, according to other case reports, this situation was also commonly found.

In conclusion, the Chiari network is a commonly found structure that may cause surgical complications, including difficulties in entering or exiting guidewires, catheters, or pacemakers. In severe cases, cardiac surgery may even be required to remove the pacemaker. Therefore, we remind surgeons to think of the Chiari network in similar situations. Echocardiography can be used to identify the Chiari network. During the surgery, forcefully pulling out a stuck guidewire is not suggested, to avoid the risk of tearing the atrial wall and causing pericardial tamponade. Urgent consultation with ultrasound doctors and cardiac surgeons might be helpful in such cases.

## Data Availability

The datasets used or analyzed during the current study are available from the corresponding author on reasonable request.

## Abbreviations

DSA: Digital subtraction angiography
